# N-fertilizer postponing application improves dry matter translocation and increases system productivity of wheat/maize intercropping

**DOI:** 10.1038/s41598-021-02345-5

**Published:** 2021-11-24

**Authors:** Ke Xu, Qiang Chai, Falong Hu, Zhilong Fan, Wen Yin

**Affiliations:** 1 College of Agronomy, Gansu Agricultural University, Lanzhou, 730070 China; 2 State Key Laboratory of Aridland Crop Science, Gansu Agricultural University, Lanzhou, 730070 China

**Keywords:** Plant sciences, Systems biology

## Abstract

Intercropping increases the grain yield to feed the ever-growing population in the world by cultivating two crop species on the same area of land. It has been proven that N-fertilizer postponed topdressing can boost the productivity of cereal/legume intercropping. However, whether the application of this technology to cereal/cereal intercropping can still increase grain yield is unclear. A field experiment was conducted from 2018 to 2020 in the arid region of northwestern China to investigate the accumulation and distribution of dry matter and yield performance of wheat/maize intercropping in response to N-fertilizer postponed topdressing application. There were three N application treatments (referred as N_1_, N_2_, N_3_) for maize and the total amount were all 360 kg N ha^−1^. N fertilizer were applied at four time, i.e. prior to sowing, at jointing stage, at pre-tasseling stage, and at 15 days post-silking stage, respectively. The N_3_ treatment was traditionally used for maize production and allocations subjected to these four stages were 2:3:4:1. The N_1_ and N_2_ were postponed topdressing treatments which allocations were 2:1:4:3 and 2:2:4:2, respectively. The results showed that the postponed topdressing N fertilizer treatments boosted the maximum average crop growth rate (CGR) of wheat/maize intercropping. The N_1_ and N_2_ treatments increased the average maximum CGR by 32.9% and 16.4% during the co-growth period, respectively, and the second average maximum CGR was increased by 29.8% and 12.6% during the maize recovery growth stage, respectively, compared with the N_3_ treatment. The N_1_ treatment was superior to other treatments, since it increased the CGR of intercropped wheat by 44.7% during the co-growth period and accelerated the CGR of intercropped maize by 29.8% after the wheat had been harvested. This treatment also increased the biomass and grain yield of intercropping by 8.6% and 33.7%, respectively, compared with the current N management practice. This yield gain was primarily attributable to the higher total translocation of dry matter. The N_1_ treatment increased the transfer amount of intercropped wheat by 28.4% from leaf and by 51.6% from stem, as well as increased the intercropped maize by 49.0% of leaf, 36.6% of stem, and 103.6% of husk, compared to N_3_ treatment, respectively. Integrated the N fertilizer postponed topdressing to the wheat/maize intercropping system have a promotion effect on increasing the translocation of dry matter to grain in vegetative organs. Therefore, the harvest index of intercropped wheat and maize with N_1_ was 5.9% and 5.3% greater than that of N_3_, respectively. This demonstrated that optimizing the management of N fertilizer can increase the grain yield from wheat/maize intercropping via the promotion of accumulation and translocation of dry matter.

## Introduction

The ever-growing population brings unprecedented challenges for agricultural production^1^. How to raise productivity and simultaneously ensure food security on the premise of environment friendliness is a thought-provoking issue. Intercropping, cultivating two or more crop species simultaneously on the same field^2^, is practiced widely throughout the world and considered to be an environment friendly system, as well as serve as a sustainable agricultural production system^3–5^. The primary reason that there are advantages to intercropping depends on the efficient use of light, nutrients, water, and other resources^3,6,7^. Research had revealed that the input of nitrogen fertilizer is the primary advantage of intercropping^8,9^. However, the application of a large amount of N causes many problems in today’s agricultural production. It is desirable to study effective theory and technology to increase food production while reducing the application of high levels of N.

The technology of reducing nitrogen application primarily includes adjusting the management of nitrogen^10^, optimizing cropping systems^11^, applying new slow/controlled fertilizers^12^, integrating water and fertilizer^13^, and applying soil conditioners^14^. Among them, optimizing the management of N in intercropping systems is a feasible technology to satisfy the requirement to decrease the application of N, while simultaneously increasing yield. The suitable management of N, designated N-fertilizer postponed topdressing, can meet the demand of maize for N to produce high yields and increase the N use efficiency^15,16^. Similarly, when applied to cereal/legume intercropping, this strategy can boost crop productivity by retarding the “inhibitory effect of N application on N_2_ fixation” by cereal crops^17^ and optimizing the intraspecific relationships^18,19^. Cereal/cereal intercropping, such as wheat/maize strip intercropping, is a long-established stable production system in northwestern China. In this system, the late-maturing crop requires more nutrients to recover its growth to eliminate the competition from crops that mature early. The use of sufficient N for the late-growth stage of cereal crops can boost the recovery growth of late-maturing crops after the harvest of species that mature early, thereby, increasing the aboveground biomass and producing a high grain yield^20^. However, a shortage of N during this time period will inhibit reproductive and vegetative development, depress the accumulation and translocation of dry matter, and lead to a decrease in yield^21,22^. Therefore, adequate N for the crop late-growth stage is essential and conducive to the accumulation of dry matter and formation of grain. It is critical that the optimization of management of N fertilizer be based on the stage of crop growth to simultaneously meet the nutrient requirements of each crop and increase the intercropping yield.

Research has shown that the accumulation and translocation of dry matter can be used to measure cultivation technologies^5,23,24^. The grain yield is commonly directly related to the transportation of photosynthetic products that are stored in vegetative organs to the reproductive organs^25,26^. Tillage^27^, irrigation^5^, row ratio^24^, and the management of N fertilizer^19,28,29^ are those measures that affect dry matter. Among them, the management N fertilizer can directly affect the efficiency of leaf photosynthetic, thus, influencing the accumulation and translocation of dry matter^30,31^. However, to our knowledge, there has been no systematic research on the transportation of photosynthetic products that have been influenced by N fertilizer postponed topdressing technology. There is a lack of effective theoretical and practical bases to improve photosynthetic products during the practice of production using this technology.

To address the aforementioned issues, a field experiment with wheat/maize intercropping was conducted to explore the effects of postponed topdressing application of N fertilizer on the distribution of dry matter and yield performance. The objectives were to (i) quantify the yield and crop growth rate of wheat and maize, (ii) determine the contribution of photosynthetic products to grain, and (iii) reveal the mechanism of yield increases through the translocation of dry matter. Our study hypothesized that the application of N fertilizer postponed topdressing to wheat/maize intercropping can increase the accumulation of photosynthesis products, improve the translocation of dry matter, and boost system productivity.

## Results

### Crop growth rate of intercropping system

**Figure 1:**
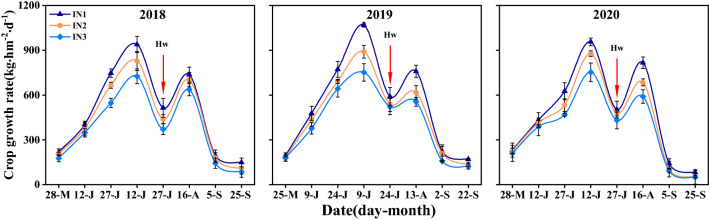
Crop growth rate of intercropping system with different N management practices in 2018–2020. I, intercropping. N_1_, N_2_, and N_3_ represent N-fertilizer applied at 51, 72, and 93 kg N ha^−1^ as first top-dressing plus 63, 42, and 21 kg N ha^−1^ at third top-dressing, respectively. Plant sampling time is 15 day intervals before wheat harvest and 20 day intervals after wheat harvest. Error bars indicates standard error of the means (*n* = 3). Arrows labeled Hw indicates wheat harvest time.

The crop growth rate (CGR) of wheat/maize intercropping system followed an obvious double-peak curve in 2018–2020 (Fig. 1). In early growth stage, there was no difference between three N-fertilizer postponing application treatments. With the growth stage development, the CGR increased markedly and reached a maximum before wheat harvest. At this stage, the averaged CGR of IN_1_ and IN_2_ treatment was 32.9% and 16.4% higher than IN_3_ treatment. Then the CGR decreased with wheat harvest. Subsequently, it reached second maximum value when maize was at early grain-filling stage. The CGR of IN_1_ and IN_2_ treatment at this stage was 29.8% and 12.6% higher than IN_3_ treatment. At final sampling time, the 3-year average CGR was increased by 56.6% with IN_1_ treatment and by 15.9% with IN_2_ treatment compared with IN_3_ treatment.

### Crop growth rate of wheat

**Figure 2:**
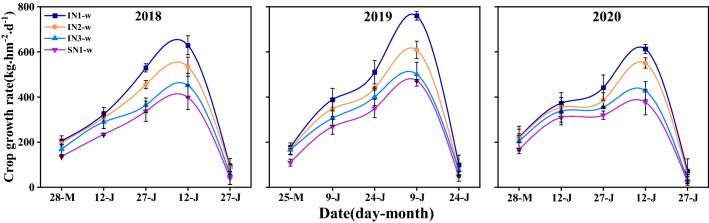
Crop growth rate of wheat in sole and intercropping systems with different N management practices in 2018–2020. I, intercropping, S, sole cropping. For intercropping system, N_1_, N_2_, and N_3_ represent N-fertilizer applied at 51, 72, and 93 kg N ha^−1^ as first top-dressing plus 63, 42, and 21 kg N ha^−1^ at third top-dressing, respectively. For sole wheat, N_1_ represents N-fertilizer applied at 108 kg N ha^−1^ as base fertilizer plus 72 kg N ha^−1^ at top-dressing. Plant sampling time is 15 day intervals before wheat harvest and 20 day intervals after wheat harvest. Error bars indicates standard error of the means (*n* = 3).

The average CGR of wheat was significantly affected by cropping system, N-fertilizer treatment, and the two factors’ interaction effect (except from early to end of May and early to end of Jul). The 3-year average CGR of intercropped wheat was higher than sole wheat in whole growth period. There was no difference between each treatment at early growth stage but this trend changed with the growth stage developed (Fig. 2). The CGR of wheat increased rapidly and reached a maximum value when wheat was at early grain-filling stage. At this stage, intercropping significantly increased it by 13.4–57.9%, 6.0–60.9% and 13.5–62.5% than sole system in 2018, 2019 and 2020, respectively. The IN_1_ and IN_2_ treatment increased it of intercropped wheat by 44.7% and 22.7% compared with IN_3_ treatment. At late growth stage, the CGR of wheat with IN_1_ and IN_2_ was 58.3% and 30.7% higher than IN_3_ treatment.

### Crop growth rate of maize

**Figure 3:**
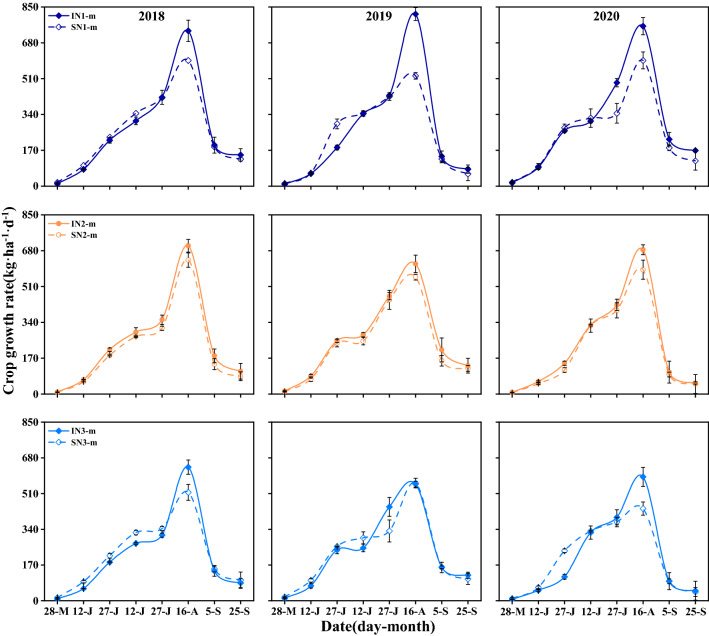
Crop growth rate of maize in sole and intercropping systems in 2018–2020 with different cropping system and N management system. I, intercropping, S, sole cropping. For intercropping system, N_1_, N_2_, and N_3_ represent N-fertilizer applied at 51, 72, and 93 kg N ha^−1^ as first top-dressing plus 63, 42, and 21 kg N ha^−1^ at third top-dressing, respectively. For sole maize, N_1_, N_2_ and N_3_ represent N-fertilizer applied at 36, 72, and 108 kg N ha^−1^ as first top-dressing plus 108, 72, and 36 kg N ha^−1^ at third top-dressing, respectively. Plant sampling time is 15 day intervals before wheat harvest and 20 day intervals after wheat harvest. Error bars indicates standard error of the means (*n* = 3).

The average CGR of maize was significantly affected by cropping system and N-fertilizer postponing application, but the two factors’ interaction effect had no influence. The growth of intercropped maize was influenced by component wheat (Fig. 3). Before wheat harvest, the CGR of maize in sole cropping was higher than that in intercropping. The SN_1-m_ and SN_2-m_ treatments increased average CGR of sole maize by 15.6–40.9% and 9.0–21.6% compared with SN_3-m_ treatment. After wheat harvest, the CGR of intercropped maize was higher than sole maize. The maximum CGR of maize was occurred at the end of July to middle of August, i.e., at anthesis to early grain filling stage. At this stage, the average CGR of intercropped maize under IN_1_ and IN_2_ treatments was increased by 29.8% and 12.6% compared with IN_3_ treatment. At the final sampling time, the IN_1_ and IN_2_ treatments improved the CGR of maize by 56.6% and 15.9% under intercropping, and by 41.6% and 12.4% under sole cropping, compared with IN_3_ treatment.

### Biomass yield of wheat and maize

**Figure 4:**
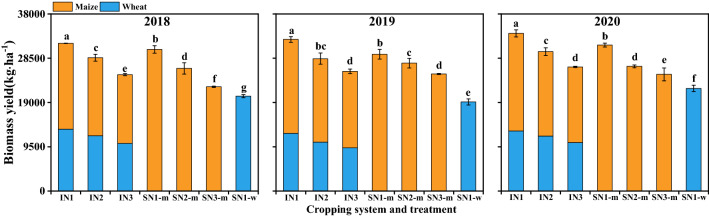
Biomass yield of wheat and maize with different N management practices under various cropping systems. I, intercropping, S, sole cropping. For intercropping system, N_1_, N_2_, and N_3_ represent N-fertilizer applied at 51, 72, and 93 kg N ha^−1^ as first top-dressing plus 63, 42, and 21 kg N ha^−1^ at third top-dressing, respectively. For sole maize, N_1_, N_2_ and N_3_ represent N-fertilizer applied at 36, 72, and 108 kg N ha^−1^ as first top-dressing plus 108, 72, and 36 kg N ha^−1^ at third top-dressing, respectively. For sole wheat, N_1_ represents the N-fertilizer applied at 108 kg N ha^−1^ as base fertilizer at sowing plus 72 kg N ha^−1^ top-dressed at pre-tasseling. Different lowercase above bars indicates significant difference (*P* ≤ 0.05) among different N managements. Error bars indicates standard error of the means (*n* = 3).

The biomass yield (BY) was significantly affected by cropping system, N-fertilizer treatment, and their interaction. On average of 3 years, the BY of intercropped wheat was 35.9–48.7% higher than that of sole wheat. The BY of intercropped maize was 12.8–31.1% higher than that of sole maize. Furthermore, the BY in intercropping was 24.7–32.9% higher than the weighted means of sole cropping (Fig. 4). For N treatment, the BY of intercropped wheat with IN_3_ was 28.7% and 14.1% lower than IN_1_ and IN_2_. Similarly, the BY of intercropped maize was 25.6% and 11.3% lower with IN_3_ compared to IN_1_ and IN_2_.

### Distribution characteristics on aboveground dry matter of wheat

The transfer amount (DTA), transfer rate (DTR), and contribution rate to grain yield (GCR) of wheat during three experimental years were significantly influenced by cropping system, but not by N treatment and their interaction (Table 1). On average, intercropping increased DTA of leaf by 65.0%, DTR by 28.2%, and GCR by 69.3% compared with sole wheat. Similarly, the DTA, DTR, and GCR of intercropped wheat were increased by 89.5%, 60.6%, and 84.6% from stem, respectively. The IN_1_ treatment increased the DTA of intercropped wheat by 28.4%, DTR by 8.1%, and GCR by 29.6% from leaf, compared with IN_3_ treatment. Similarly, the DTA, DTR, and GCR were increased by 51.6%, 34.1%, and 55.0% from stem, respectively. Furthermore, the IN_2_ treatment increased the DTA of intercropped wheat by 10.7%, DTR by 3.4%, and GCR by 11.6% from leaf; and by 14.9%, 10.6%, and 19.9% from stem, compared with IN_3_ treatment.

**Table 1:** Dry matter translocation and contribution rate to grain yield of leaf and stem in wheat in intercropping and sole cropping systems under different N management.

Year	Cropping system^a^	N management practice^b^	Leaf			Stem		
			DTA^c^/kg	DTR/%	GCR/%	DTA/kg	DTR/%	GCR/%
2018	Intercrop	IN_1_	1823a	60.4a	21.1a	1350a	12.8abcd	15.7a
		IN_2_	1598ab	58.7ab	19.56a	1238a	12.6abcd	15.1ab
		IN_3_	1508ab	57.8abc	18.7a	1058ab	11.6abcde	13.2ab
	Sole crop	SN_1-w_	810d	34.2f	11.9bc	743bcd	10.2bcde	10.9abc
2019	Intercrop	IN_1_	1845a	49.5bcde	19.5a	1328a	15.1ab	14.2ab
		IN_2_	1620ab	48.1de	17.1ab	607cd	8.2cde	6.5cde
		IN_3_	1440ab	47.2de	16.5ab	540cd	7.8de	6.2cde
	Sole crop	SN_1-w_	1148bcd	45.4de	11.6bc	360d	5.9e	3.7e
2020	Intercrop	IN_1_	1596ab	52.7abcd	21.0a	1187a	16.3a	15.6a
		IN_2_	1321bc	48.8cde	16.6ab	1084ab	15.7ab	13.6ab
		IN_3_	1151bcd	45.4de	12.4bc	950abc	13.6abc	10.0bcd
	Sole crop	SN_1-w_	851cd	42.1ef	8.4c	540cd	7.5de	5.2de
**Significance (*p* value)**								
Cropping system (C)			0.000	0.000	0.000	0.000	0.000	0.000
N management practice (N)			0.092	0.063	0.671	0.248	0.126	0.133
C × N			0.092	0.063	0.671	0.248	0.126	0.133

Means with the same lowercase letters in the same column are significantly different at *P* ≤ 0.05.

^a^Intercrop and sole crop means the intercropped wheat and sole wheat.

^b^For intercropping system, N_1_, N_2_, and N_3_ represent N-fertilizer applied at 51, 72, and 93 kg N ha^−1^ as first top-dressing plus 63, 42, and 21 kg N ha^−1^ at third top-dressing, respectively. For sole cropping, N_1_ represents N-fertilizer applied at 108 kg N ha^−1^ as base fertilizer plus 72 kg N ha^−1^ at top-dressing.

^c^DTA is transportation amount of dry matter in vegetative organ (kg); DTR is transfer rate of dry matter in vegetative organ (%); GCR is contribution rate of vegetative organs to grain (%).

### Distribution characteristics on aboveground dry matter of maize

The DTA, DTR, and GCR of maize were significantly influenced by the cropping system and N treatment, but not by their interaction (Table 2). On average, intercropping increased the DTA by 38.7%, DTR by 29.1%, and GCR by 53.6% from leaf, compared with sole maize. Similarly, DTA, DTR, and GCR were increased by 27.4%, 20.4%, and 40.6% from stem, and by 51.4%, 61.2%, and 64.5% from husk, respectively. In wheat/maize intercropping, the IN_1_ treatment increased the DTA of leaf by 49.0%, DTR by 32.6%, and GCR by 48.4% compared to IN_3_ treatment. Similarly, DTA, DTR, and GCR were increased by 36.6%, 8.6%, and 39.1% from stem, and increased by 103.6%, 36.8%, and 105.7% from husk, respectively. In addition, the IN_2_ treatment increased the DTA, DTR and GCR by 19.1%, 13.2%, and 12.6% from leaf, 14.3%, 5.3%, and 10.6% from stem and 43.6%, 19.7%, and 36.1% from husk compared with IN_3_ treatment.

**Table 2:** Dry matter translocation and contribution rate to grain yield of leaf, stem, and husk in maize of intercropping and sole cropping systems under different N management.

Year	Cropping system^a^	N management practice^b^	Leaf			Stem			Husk		
			DTA^c^/kg	DTR/%	GCR/%	DTA/kg	DTR/%	GCR/%	DTA/kg	DTR/%	GCR/%
2018	Intercrop	IN_1_	1371abc	27.7ab	8.04ab	2116ab	27.5ab	12.4ab	990a	21.0abc	5.79a
		IN_2_	1177cde	25.1abc	6.28cdef	1869abcd	26.4abc	10.0abcd	786abc	19.9abcd	4.19abc
		IN_3_	998efg	21.4abcd	6.16cdef	1528cdef	25.0abcd	9.5abcd	530cdef	16.2bcdef	3.28bcd
	Sole crop	SN_1-m_	1293bcd	24.7abc	6.87bcde	1701bcdef	22.8abcd	9.0bcd	888ab	19.8abcd	4.71ab
		SN_2-m_	935fgh	18.7cde	4.94defg	1458cdef	21.6abcd	7.9cde	564cde	14.5cdef	3.12bcd
		SN_3-m_	630gh	13.7de	3.10gh	1286ef	20.8bcd	6.7de	353efg	10.8f	1.73def
2019	Intercrop	IN_1_	1471ab	28.9a	7.80abc	2317a	28.6a	12.2ab	911ab	18.0abcde	4.82ab
		IN_2_	1097def	21.9abc	5.39defg	1915abcd	28.1a	9.5abcd	516cdef	13.7def	2.53cde
		IN_3_	960fgh	19.0bcde	4.60efgh	1781bcde	26.0abc	8.6bcde	266fg	9.55fg	1.28ef
	Sole crop	SN_1-m_	1156cde	22.4abc	4.33efgh	1961abc	23.3abcd	7.6cde	550cdef	14.0cdef	2.08def
		SN_2-m_	905fgh	19.1cde	3.85fgh	1635bcdef	20.0cd	7.0cde	452def	12.1ef	1.92def
		SN_3-m_	533h	12.6e	1.94h	1288ef	18.0d	4.7e	117g	3.58g	0.42f
2020	Intercrop	IN_1_	1593a	27.9ab	9.04a	2341a	24.2abcd	13.5a	938ab	24.6a	5.56a
		IN_2_	1271cde	25.2abc	7.22abcd	1883abcd	23.3abcd	10.9abc	701bcd	22.0ab	3.98abc
		IN_3_	1018efg	22.5abc	6.00cdefg	1650bcdef	23.0abcd	9.4bcd	598cde	20.7abcd	3.30bcd
	Sole crop	SN_1-m_	1108def	23.3abc	7.25abcd	1715bcdef	24.0abcd	10.9abc	525cdef	12.1ef	3.41bcd
		SN_2-m_	835fgh	19.5bcde	4.39efgh	1406def	22.2abcd	7.5cde	450def	11.2ef	2.39cde
		SN_3-m_	710fgh	18.8cde	4.15efgh	1229f	20.4bcd	7.2cde	335efg	9.05fg	1.96def
**Significance (*p* value)**											
Cropping system (C)			0.000	0.000	0.000	0.000	0.000	0.000	0.000	0.000	0.000
N management system(N)			0.000	0.000	0.000	0.000	0.064	0.000	0.000	0.001	0.000
C × N			0.819	0.834	0.819	0.865	0.798	0.794	0.585	0.805	0.469

Means with the same lowercase letters in the same column are significantly different at *P* ≤ 0.05.

^a^Intercrop and sole crop means the intercropped maize and sole maize.

^b^For intercropping, N_1_, N_2_, and N_3_ represent N-fertilizer applied at 51, 72, and 93 kg N ha^−1^ as first top-dressing plus 63, 42, and 21 kg N ha^−1^ at third top-dressing, respectively. For sole cropping, N_1_, N_2_ and N_3_ represent N-fertilizer applied at 36, 72, and 108 kg N ha^−1^ as first top-dressing plus 108, 72, and 36 kg N ha^−1^ at third top-dressing, respectively.

^c^DTA is transportation amount of dry matter in vegetative organ (kg); DTR is transfer rate of dry matter in vegetative organ (%); GCR is contribution rate of vegetative organs to grain (%).

### Grain yield of wheat and maize

**Figure 5:**
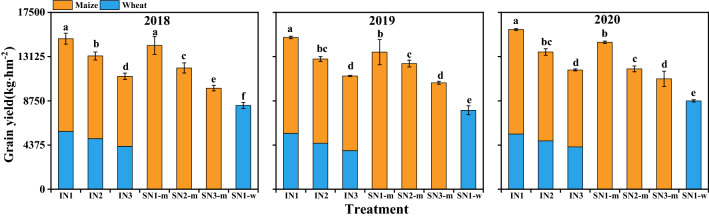
Grain yield of wheat and maize in sole and intercropping systems as affected by N management practices in 2018–2020. I, intercropping, S, sole cropping. For intercropping system, N_1_, N_2_, and N_3_ represent N-fertilizer applied at 51, 72, and 93 kg N ha^−1^ as first top-dressing plus 63, 42, and 21 kg N ha^−1^ at third top-dressing, respectively. For sole maize, N_1_, N_2_ and N_3_ represent N-fertilizer applied at 36, 72, and 108 kg N ha^−1^ as first top-dressing plus 108, 72, and 36 kg N ha^−1^ at third top-dressing, respectively. For sole wheat, N_1_ represents the N-fertilizer applied at 108 kg N ha^−1^ as base fertilizer at sowing plus 72 kg N ha^−1^ top-dressed at pre-tasseling. Different lowercase above bars indicates significant difference (*P* ≤ 0.05) among different N managements. Error bars indicates standard error of the means (*n* = 3).

Copping system and N treatment individually had a significant effect on grain yield (GY) of wheat and maize in each year, and their interaction did as well (Fig. 5). It was consistent that crops in the intercropping system had yield advantages compared to corresponding sole crops. The GY in intercropping was 19.1–30.7% higher than the weighted means of sole cropping. In intercropping system, IN_1_ and IN_2_ increased the mixed yield by 33.3% and 18.0% in 2018, 34.1% and 14.9% in 2019, and 33.8% and 15.0% in 2020, compared with IN_3_ treatment, and IN_1_ treatment exhibited the most significant effect in improving grain yield. The GY of sole maize with SN_1-m_ and SN_2-m_ were 42.1% and 19.9% greater than SN_3-m_ in 2018, 28.9% and 18.0% in 2019, and 33.0% and 8.9% in 2020.

### Land use efficiency

**Figure 6:**
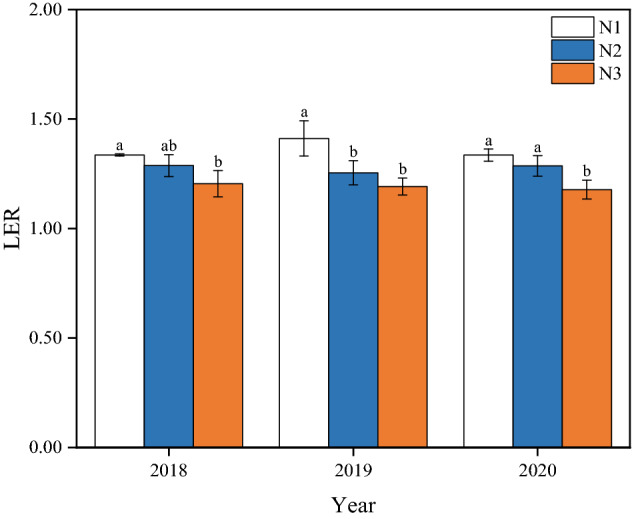
Land use efficiency of wheat/maize intercropping systems under different N managements practices in 2018–2020. N_1_, N_2_, and N_3_ represent N-fertilizer applied at 51, 72, and 93 kg N ha^−1^ as first top-dressing plus 63, 42, and 21 kg N ha^−1^ at third top-dressing, respectively.

The total LER of the wheat/maize intercropping was greater than 1.0 (Fig. 6). On average, the LER under N_1_ treatment was 6.6% and 7.0% higher than N_2_ and N_3_ treatments, respectively. Meantime, the LER of N_2_ treatment was 7.0% higher than N_3_ treatment.

### Yield components of wheat

The yield components are significant factors for achieving high yield of crops. Cropping system had a significant influence on spike number (SN) and thousand-kernel weight (TKW) of wheat, but not by N management and their interaction (Table 3). The SN of intercropped wheat was 11.9–25.2% higher than that of sole wheat under the same land area. Similarly, the KNS of intercropped wheat was 5.4–18.3% higher than sole wheat. Whereas, the TKW were 6.9–14.7% lower than sole wheat. The SN with the IN_1_ and IN_2_ were increased by 11.9 and 4.5%; KNS were increased by 16.0% and 10.0%; and TKW were increased by 7.3% and 3.2%, compared with IN_3_, respectively.

**Table 3:** The spike number (SN), kernel number per spike (KNS), and thousand-kernel weight (TKW) of wheat in sole crop and intercrop as affected by cropping system and N management in 2018–2020.

Cropping systems^a^	N management system^b^	SN 10^4^ (ha^−1^)			KNS			TKW(g)		
		2018	2019	2020	2018	2019	2020	2018	2019	2020
Intercrop	IN_1_	327b	323b	341b	22.0a	38.6a	35.8a	43.6ab	42.9b	44.8b
	IN_2_	308b	298b	320b	20.8a	35.4b	35.3a	42.0bc	41.9b	42.3c
	IN_3_	298b	283b	305b	18.0a	33.5c	31.6a	40.7c	40.7b	40.9c
Sole crop	SN_1-w_	623a	603a	655a	16.9a	34.2bc	29.2a	45.6a	47.5a	47.2a
**Significance (*p* value)**										
Cropping system (C)		0.000	0.000	0.000	0.341	0.001	0.228	0.000	0.000	0.000
N management system(N)		0.907	0.793	0.826	0.888	0.000	0.898	0.152	0.586	0.044
C × N		0.907	0.793	0.826	0.888	0.000	0.898	0.152	0.586	0.044

Means with the same lowercase letters in the same column are significantly different at *P* ≤ 0.05.

^a^Intercrop and sole crop means the intercropped wheat and sole wheat.

^b^For intercropping, N_1_, N_2_, and N_3_ represent N-fertilizer applied at 51, 72, and 93 kg N ha^−1^ as first top-dressing plus 63, 42, and 21 kg N ha^−1^ at third top-dressing, respectively. For sole cropping, N_1_ represents N-fertilizer applied at 108 kg N ha^−1^ as base fertilizer plus 72 kg N ha^−1^ at top-dressing.

### Yield components of maize

The SN, and TKW of maize were significantly affected by cropping system, but not by N management and their interaction (Table 4). The SN of intercropped maize was 6.5–15.7% higher than that of sole maize under the same land area. Whereas, the KNS and TKW of intercropped maize was were 6.2–11.1% and 11.1–17.6% lower than sole maize. The SN of sole maize with the SN_1-m_ and SN_2-m_ were promoted by 8.5% and 7.0%, the KNS by 14.9% and 5.7%, and TKW by 5.7% and 3.5% compared with SN_3-m_ treatment, respectively. The same trend was found in intercropping maize. The SN of intercropped maize under IN_1_ and IN_2_ were 24.8% and 15.5% higher than IN_3_, the KNS were 12.0% and 7.7%, and TKW were 11.9% and 9.5%, respectively.

**Table 4:** The spike number (SN), kernel number per spike (KNS), and thousand-kernel weight (TKW) of maize in sole crop and intercrop as affected by cropping system and N management in 2018–2020.

Cropping systems^a^	N management system^b^	SN 10^4^ (ha^−1^)			KNS			TKW(g)		
		2018	2019	2020	2018	2019	2020	2018	2019	2020
Intercrop	IN_1_	6.89c	7.45bc	7.06c	564ab	543ab	472a	347abc	362b	360b
	IN_2_	6.06cd	6.52c	6.00d	550ab	517ab	452a	334bc	356b	359ab
	IN_3_	5.17d	5.84c	5.61d	481c	479c	449a	310c	326c	320ab
Sole crop	SN_1-m_	10.18a	10.96a	10.78a	648a	565a	541a	394a	402a	393a
	SN_2-m_	9.29a	10.15a	9.39b	572ab	551ab	489a	389a	395a	381a
	SN_3-m_	8.27b	9.72ab	9.00b	551ab	499ab	475a	374ab	381ab	370a
**Significance (*p* value)**										
Cropping system (C)		0.000	0.002	0.000	0.132	0.238	0.301	0.001	0.000	0.006
N management system(N)		0.000	0.192	0.000	0.166	0.061	0.649	0.243	0.018	0.064
C × N		0.946	0.967	0.798	0.769	0.959	0.904	0.877	0.615	0.554

Means with the same lowercase letters in the same column are significantly different at *P* < 0.05.

^a^Intercrop and sole crop means the intercropped maize and sole maize.

^b^For intercropping, N_1_, N_2_, and N_3_ represent N-fertilizer applied at 51, 72, and 93 kg N ha^−1^ as first top-dressing plus 63, 42, and 21 kg N ha^−1^ at third top-dressing, respectively. For sole cropping, N_1_, N_2_ and N_3_ represent N-fertilizer applied at 36, 72, and 108 kg N ha^−1^ as first top-dressing plus 108, 72, and 36 kg N ha^−1^ at third top-dressing, respectively.

### Harvest index of wheat and maize

**Figure 7:**
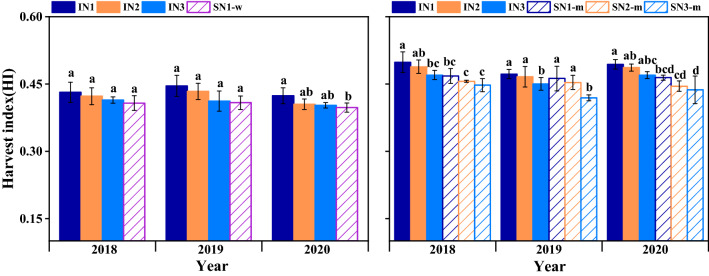
Harvest index of wheat and maize in sole and intercropping systems with different N management practices in 2018–2020. I, intercropping, S, sole cropping. For intercropping system, N_1_, N_2_, and N_3_ represent N-fertilizer applied at 51, 72, and 93 kg N ha^−1^ as first top-dressing plus 63, 42, and 21 kg N ha^−1^ at third top-dressing, respectively. For sole maize, N_1_, N_2_ and N_3_ represent N-fertilizer applied at 36, 72, and 108 kg N ha^−1^ as first top-dressing plus 108, 72, and 36 kg N ha^−1^ at third top-dressing, respectively. For sole wheat, N_1_ represents the N-fertilizer applied at 108 kg N ha^−1^ as base fertilizer at sowing plus 72 kg N ha^−1^ top-dressed at pre-tasseling. Different lowercase above bars indicates significant difference (*P* ≤ 0.05) among different N managements. Error bars indicates standard error of the means (*n* = 3).

Harvest index (HI) of wheat and maize was significantly affected by cropping system and N-fertilizer treatment (except for wheat), but not by their interaction (Fig. 7). The HI of intercropped wheat with IN_1_ and IN_2_ was 5.9% and 2.6% greater than IN_3_, and of intercropped maize was 5.3% and 3.6% with IN_1_ and IN_2_ compared with IN_3_. The HI of sole maize with SN_1-m_ and SN_2-m_ was 6.9% and 3.9% higher than that of SN_3-m_. The HI of sole wheat was lowest, only reached to 0.40. Among the three N treatments, IN_1_ and IN_2_ increased the HI of intercropped wheat and maize.

### Path analysis

**Figure 8:**
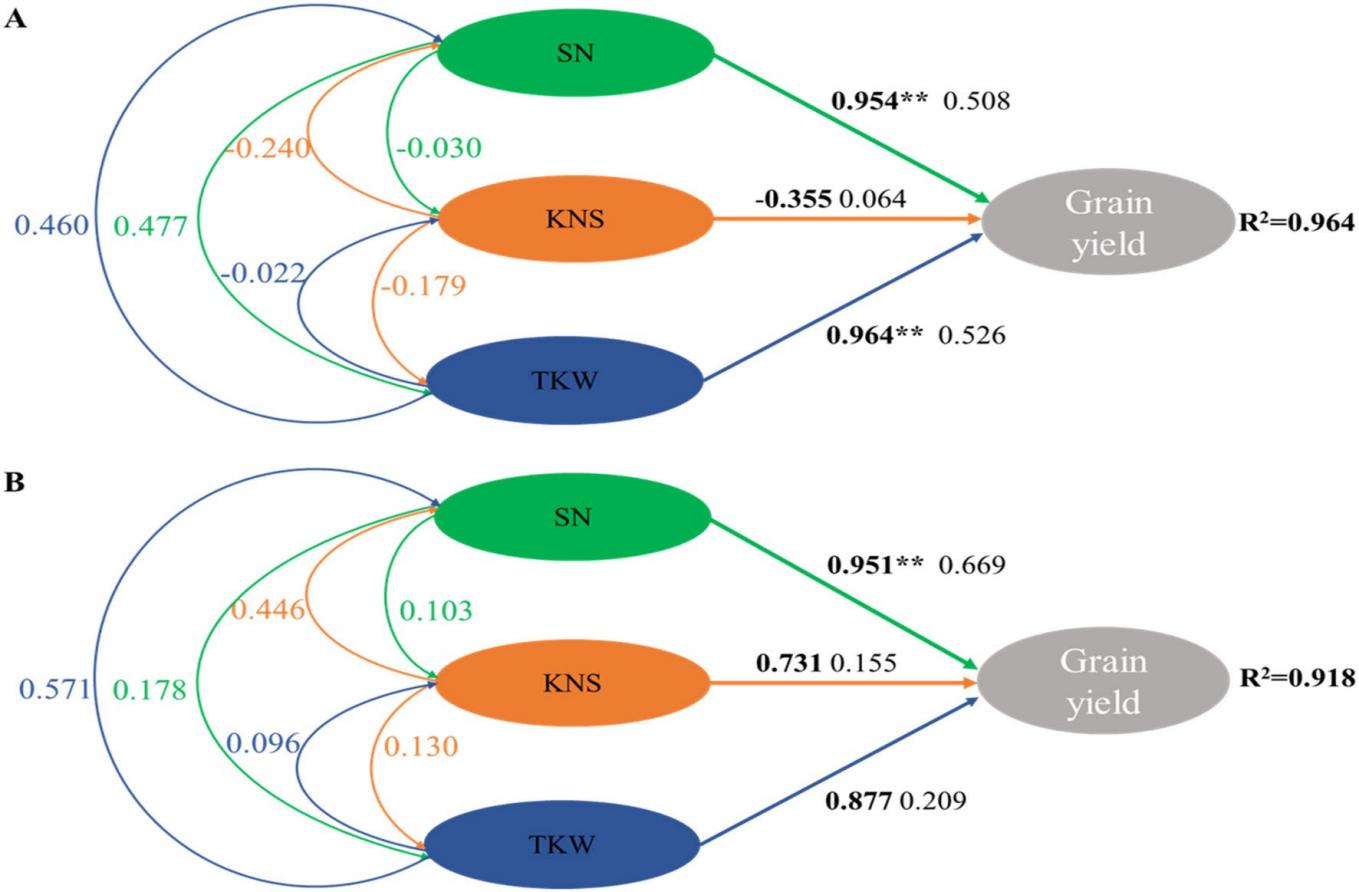
Overall path analysis of yield components for wheat (**A**) and maize (**B**) grain yield with thick lines represent direct pathways and fine lines represent indirect pathways. Values in bold are the correlation coefficient and fine values are the path coefficient. *P ≤ 0.05, **P ≤ 0.01. SNE, KNS and TKW indicate the spike number, kernel number per spike, and thousand-kernel weight, respectively.

The correlation coefficients between the grain yield and yield components were used to separate into direct and indirect effects via path analysis (Fig. 8A). The spike number (SN) and thousand-kernel weight (TKW) of wheat had the highest direct path coefficient and correlation coefficient than kernel number per spike (KNS). In addition, TKW had a positive indirect path coefficient with SN and SN had a positive indirect path coefficient with TKW, indicating that yield was influenced by the interaction between them. Although KNS has the lowest direct path coefficient (0.064), the indirect path coefficient of KNS to SN is − 0.240, which is 3.75 times for its direct path coefficient.

The SN of maize was significantly correlated with grain yield (Fig. 8B). Furthermore, SN had the highest the correlation coefficient than TKW and KNS, indicating SN had direct influence on grain yield. Nevertheless, KNS and TKW could indirectly affect grain yield via SN, with TKW contributing more than KNS.

## Discussion

### The crop growth rate and biomass yield

Numerous studies had reported one important factor affecting the obtain of grain yield was dry matter accumulation^32,33^. This is mainly because dry matter accumulation and distribution in reproductive organs of crops, can reflect indirectly grain yield response to the availability of resource^34^. In the present study, the CGR of wheat/maize intercropping presents an obvious double-peak curve in each studied year. Before wheat harvest, the CGR of intercropping reached the maximum value. After wheat harvest, it decreased and reached the second maximum value when maize was at early grain filling stage. The reason was that intercropped wheat was earlier planted and created a competitive advantage over the later planted intercropped maize for resources uptake during co-growth period, resulted in a strong suppression of intercropped maize^5,9^. Thus, the CGR of the intercropped wheat was higher than sole wheat. Owing to the high light intensity, wheat (C3 crop) may use light more efficiently in the intercropping than in sole crop during co-growth period^35,36^. The CGR of intercropped maize was lower than sole cropping before wheat harvest, but higher after wheat harvest. This result was consistent with previous studies, with aboveground dry matter of maize showing recovery growth after wheat harvest^5^. Therefore, the weighted means of BY in intercropping was 24.7–32.9% higher than that of monocropping. That means the intercropping can accumulate more dry matter than the corresponding sole system.

In this study, IN_1_ and IN_2_ treatment, which postponed 20% and 10% of total N fertilizer from maize jointing stage to 15 days post-silking stage, had a significant effect on boosting the maximum CGR of intercropped wheat, which was boosted by 44.7% and 22.7% compared to IN_3_ treatment. That may because the IN_3_ treatment used excessive N fertilizer at maize jointing stage which is not suitable for intercropped wheat growing. Numerous studies have shown that nutrients play a crucial role in recovery growth of late-maturing crops after the early-maturing crops harvest^11,20,37^. Compared to the IN_3_ treatment, the IN_1_ and IN_2_ treatment increased the CGR of intercropped maize by 29.8% and 12.6%. The reason might be the postponed topdressing N fertilizer greatly intensified the interspecific competition in co-growth period but eventually generated a substantial complementarity^19^. This was similar to previous research that adequate N supply plays a pivotal role in recovery growth of intercropped maize after wheat harvest^20^.

### The transfer of vegetative products to ear

The proportion of photosynthetic products stored in leaves and stems is relatively small and most dry matter accumulation during the grain-filling period is accumulated in grain^33^. In this study, intercropping had a significant effect on aboveground dry matter translocation. It increased DTA of leaf by 65.0%, DTR by 28.2%, and GCR by 69.3% and 89.5%, 60.6%, and 84.6% from stem compared with sole wheat, respectively. One main reason is that during the co-growth period, wheat has a competitive advantage, and can obtain more light and heat resources. Meantime, wheat shaded the adjacent maize, thus reducing solar radiation received by maize^5,38^. Interspecific competition not only includes aboveground competition but also contains belowground competition. Belowground competition was mainly for growth space, water, and nutrients. As shown in this study, IN_1_ and IN_2_ treatments increased the DTA of intercropped wheat by 10.7–28.4%, DTR by 3.4–8.1%, and GCR by 11.6–29.6% from leaf, compared to IN_3_ treatment, and 14.9–51.6%, 10.6–34.1%, 19.9–55.0% from stem, respectively. Therefore, IN_1_ treatment showed the best effect on optimizing dry matter distribution of aboveground tissue in intercropped wheat. Previous research suggested that adequate N supply directly affects the production, partitioning, and translocation of dry matter^22^. An increasing in wheat transferring amount, transferring rate, and contribution rate to grain might because wheat has a higher competitive ability for N. Intercropped wheat having much greater root length density, and roots spreading laterally into the maize strip during the co-growth period^39^, and then competing for N from the adjacent maize strip.

However, late-maturing crops could form the compensatory effect of time and space when early-maturing crops were harvested. In this study, intercropping increased the DTA by 38.7%, DTR by 29.1%, and GCR by 53.6% from leaf compared to sole maize, by 27.4%, 20.4%, and 40.6% from stem, and by 51.4%, 61.2%, and 64.5% from husk, respectively. That means the increasing in maize aboveground dry matter translocation probably resulted from compensatory effect, which late-maturing crops (such as maize and soybean) root gradually expand to the underground space of early-maturing crops (like wheat) after it harvest, absorb more nutrient and water, thereby accelerated the growth rate of late-maturing crops^11^. It has been confirmed that recovery growth is fundamentally related to the supplemental N^40^. In wheat/maize intercropping, the IN_1_ treatment increased the DTA, DTR, and GCR by 49.0%, 32.6%, and 48.4% from leaf, by 36.6%, 8.6%, and 39.1% from stem, and by 103.6%, 36.8%, and 105.7% from husk compared to IN_3_ treatment, respectively. In this study, maize performed the highest compensatory intensity during the third recovery stage (i.e., from grain filling to maturity), which was similar to previous research^41^. That is to say, suitable fertilizer N management at this stage is the key to enhance recovery growth. The IN_1_ treatment transferred 20% of total N at this stage can well match fertilizer N supply with crop N requirement.

### Yield performance and yield components

The common advantages of intercropping are (i) efficient use of nutrients, light, and water^42,43^, (ii) achieving agricultural biodiversity, and (iii) increasing yield^28,44^. In northwest China, wheat/maize intercropping, an old cropping practice that aims to match efficient crop demands to the available growth resources and labor, has been widely used by farmers^45^. In the present study, the grain yield in intercropping was 19.1–30.7% higher than the weighted means of corresponding sole cropping. It was because intercropped wheat had a strong competition relative to the accompanying maize, more resources in the adjacent vacant area were available to intercropped wheat^28^, thus intercropped wheat obtained greater yield components and higher grain yield than sole wheat under the same area. After wheat harvest, expansion of absorption space for light, heat, and gas resources on the ground coupled with the expansion of absorption scope for water and nutrients underground gave intercropped maize a chance to compensate, which is the basis for high yields of intercropped maize^11^. It has been discovered that coordinated development among yield components is the foundation for achieving high grain yield for cereal crops^46,47^. In present study, intercropping increased the yield components of wheat and maize. Under the same land area, intercropping with the three N fertilizer postponed topdressing treatment increased SN of wheat by an average 18.0% and by 11.2% of maize compared to sole cropping, across the 3 years. Similarly, intercropping increased KNS of wheat by 15.0%. This is mainly because that favorable interspecific competition and compensation effect is beneficial to improve yield components and crop grain yield, thus obtaining the higher harvest index^47^.

In present study, IN_1_ and IN_2_ treatments boosted the mixed yield by 33.7% and 15.9% compared with IN_3_ treatment. It had been reported that the N_1_ treatment, where 45 kg N ha^−1^ was applied at the first topdressing plus 135 kg N ha^−1^ at the third topdressing, can boost the grain yield of intercropped pea and maize compared to the N_3_ treatment which 135 kg N ha^−1^ was topdressing at the first plus 45 kg N ha^−1^ at the third topdressing^19^. Mainly because the competitive ability of legumes was improved in planting mixtures so as to enhance the yield of intercropping^48^. N application could not only boost the grain numbers per unit areas, but also improve grain protein concentration^49^. The IN_1_ and IN_2_ treatment increased the spike number (by 13.8 and 5.0%), kernel number per spike (by 16.0% and 10.0%), and the thousand-kernel weight (by 7.3% and 3.2%) of intercropped wheat; similarly enhanced the spike number (by 24.8% and 15.5%), the kernel number per spike (by 12.0% and 7.7%), and the thousand-kernel weight (by 11.9% and 9.5%) of intercropped maize, respectively. One reason for this phenomenon might be N-fertilizer postponed topdressing is an effective approach to match fertilizer N supply with crop N requirement which is crucial to achieving high productivity^10^. Path analysis showed that grain yield of wheat was mainly derived from spike number and thousand-kernel weight, and while kernel number per spike indirectly influences spike number so as to affect the grain yield. The grain yield of maize was mainly derived from spike number, while thousand-kernel weight and while kernel number per spike indirectly influences spike number so as to affect the grain yield. In this experiment, IN_1_ and IN_2_ treatments increased the average HI of intercropped wheat by 5.9% and 2.6%, and by 5.3% and 3.6% of intercropped maize compared to IN_3_ treatment. This mainly because intercropped wheat can capture more resources during the co-growth stage and intercropped maize attributed to more transfer of aboveground dry matter to vegetative organs to ear during the late-growth stage^50^. Furthermore, the total LER of wheat/maize intercropping averages 1.28, which indicated the intercropping system used less land but produced more grain than their corresponding monocultures. This means intercropping system can more efficiently use the resources to product than monocultures.

## Conclusions

The N-fertilizer postponed topdressing treatments, which transferred 20% or 10% of the total amount N from the jointing stage to 15 days post-silking stage, boosted the crop growth rate of intercropping wheat during the co-growth stage and simultaneously accelerated the crop growth rate of intercropping maize crops during their recovery growth stage, respectively. They also increased the biomass yield of intercropping by 8.6% and 5.0%, compared with traditional N management practices, respectively. The N fertilizer postponed topdressing optimized the transfer of dry matter from vegetative organs to grain and increased the proportion postponed that boosted the amount of transportation. The postponed topdressing applications at 20% and 10% enhanced the mixed grain yield by 33.7% and 16.0%, compared with traditional N management practices, respectively. The harvest index of intercropped wheat increased by 5.9% and 2.6%, respectively, and that of intercropped maize by 5.3% and 3.6%, compared with traditional N management practices, respectively. Our results showed that N fertilizer postponed topdressing, particularly postponing the application at 20%, can increase the accumulation of photosynthetic products and optimize the translocation of dry matter, which improved the productivity of intercropping systems.

## Materials and methods

### Test site description

The field experiment was carried out in 2018–2020 at the Oasis Agricultural Trial Station (37°30′N, 103°5′E; 1776 m a.s.l.) of Gansu Agricultural University. The station is located in the eastern part of the Hexi Corridor of northwestern China. At experimental site, the average annual sunshine duration (1960–2009) was 2945 h, annual air temperature was 7.2 °C, and accumulated temperature (above 10 °C) was 2985 °C. In this region, the accumulated heat and light is abundant for one crop per year but insufficient for two, which is suitable for developing of intercropping. Wheat/maize intercropping, introduced to this region since the twentieth century, is still a prevailing cropping system^44^. The soil at the experimental site is classified as an Aridisol^51^. Before the experiment, soil properties of the top 0–30 cm soil layer were 8.0 pH (1:2.5 soil:water) using a pH meter, 11.3 g/kg soil organic carbon by the potassium dichromate heating oxidation-volumetric method, 1.44 g/cm^3^ soil bulk density by cutting ring method, 0.94 g/kg total N by Elementar (Vario MACRO cube, Germany), 29.2 mg/kg available phosphorous (P; Olsen-P) by the molybdenum-blue method, and 152.6 mg/kg available potassium (K; NH_4_OAc-extractable-K) by NH4OAc soaking method.

### Experimental design

**Figure 9:**
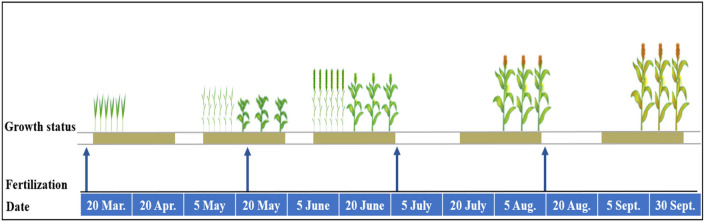
Main growth stages of wheat/maize intercropping, and time of fertilization at the experimental site in northwestern China.

The experimental design was a factorial design with seven treatments and three replications. Cropping systems were sole maize, sole wheat, and wheat/maize intercropping. Three N-fertilizer postponed top-dressing treatments (N_1_, N_2_ and N_3_) were designed according to key growth stage of maize that was jointing stage (V6), pre-tasseling stage (V12), and 15 days post-silking (R2) (Fig. 9). The N_3_ treatment is the local N management practice in this region. N fertilizer rate for sole maize was 360 kg N ha^−1^, in which 20% and 40% of total N application were applied pre-plant and top-dressed at pre-tasseling stage, respectively. The remaining 40% was divided into jointing stage and 15 days post-silking stage and the allocations were, respectively: 10% and 30% for N_1_; 20% and 20% for N_2_; and 30% and 10% for N_3_, thus formed postponing application of 20% (N_1_), 10% (N_2_), and without postponing application (N_3_). The total amount of N fertilizer was 285 kg N ha^−1^ for wheat/maize intercropping, which was calculated by the bandwidth ratio. N fertilizer rate for sole wheat was 180 kg N ha^−1^, in which 108 kg N ha^−1^ was base applied at sowing and 72 kg N ha^−1^ at booting stage (i.e. pre-tasseling stage of maize). Crops in sole and intercropping received an equivalent N rate at specific area. The detailed treatment code and N-fertilizer management were presented in Table 5. The amount of phosphorus was 180 kg P ha^−1^ and applied in all plots before sowing. The types of N and P were urea (46–0–0, N–P–K) and superphosphate (11–51–0) fertilizers. The topdressing fertilizer in maize strips was achieved by the drip irrigation method.

**Table 5:** N fertilizer allocation amount (kg ha^−1^) and percentage in each treatment.

Cropping systems	N management system^a^	Base N fertilizer^b^	Top-dressing of N fertilizer			Postponed percentage^c^ (%)	N fertilizer Total
			Jointing	Pre-tasseling	15 days post-silking		
Wheat/maize intercropping	IN_1_	88	51	83	63	20	285
	IN_2_	88	72	83	42	10	285
	IN_3_	88	93	83	21	–	285
Sole maize	SN_1-m_	72	36	144	108	20	360
	SN_2-m_	72	72	144	72	10	360
	SN_3-m_	72	108	144	36	–	360
Sole wheat	SN_1-w_	108	72	0	0	–	180

^a^For sole maize, N_1_, N_2_ and N_3_ represent N-fertilizer applied at 36, 72, and 108 kg N ha^−1^ as first top-dressing plus 108, 72, and 36 kg N ha^−1^ at third top-dressing, respectively. For sole wheat, N_1_ represents the N-fertilizer applied at 108 kg N ha^−1^ as base fertilizer at sowing plus 72 kg N ha^−1^ top-dressed at pre-tasseling.

^b^Intercropped components (i.e., maize and wheat) received the same area-based N fertilizer rate as the corresponding sole crops.

^c^The postponed percentage applied only for maize.

**Figure 10:**
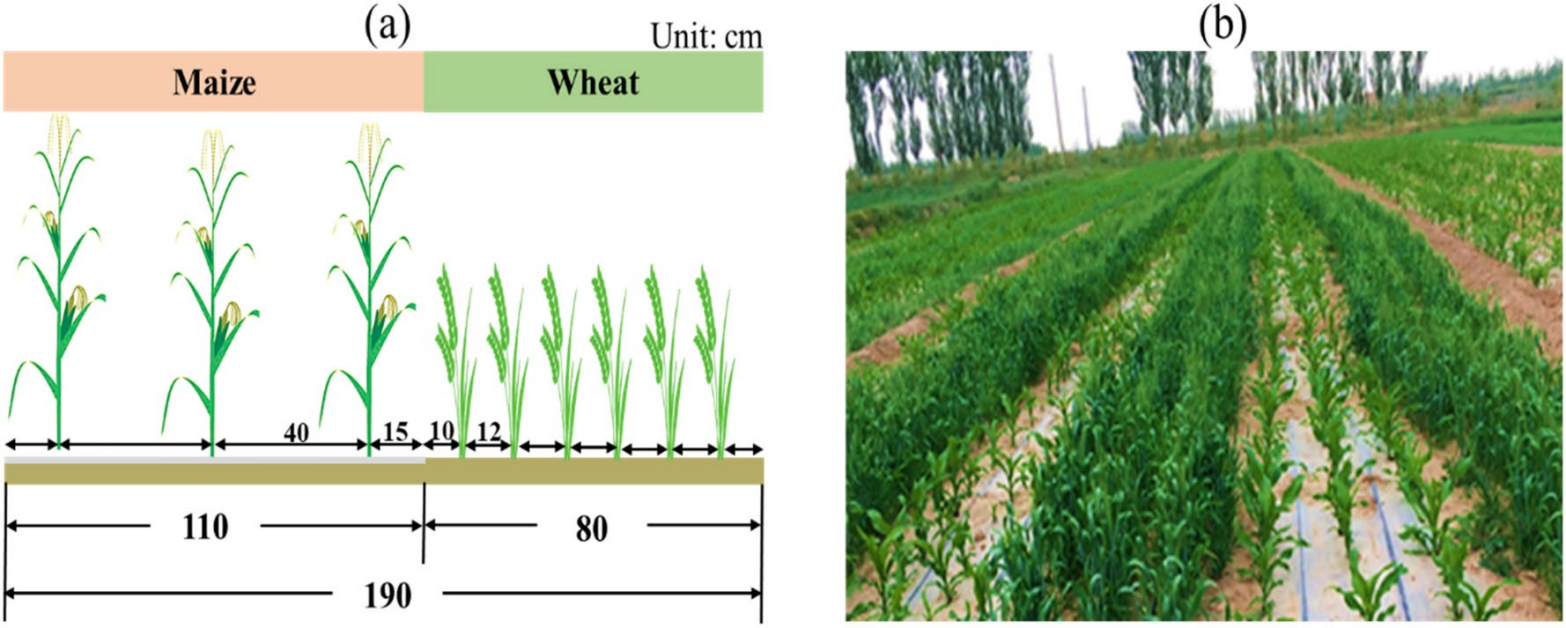
Information of (**a**) the spatial arrangement of wheat/maize intercropping with wheat strip of 80-cm (six rows) alternated with maize strip of 110-cm (three rows) and (**b**) the field planting diagrammatic representation of wheat/maize intercropping at Wuwei experimental station in northwestern China.

The plot size for intercropping was 5.7 m length × 6 m width, and for sole cropping was 6 m length × 6 m width, with every neighboring plot had a 50 cm wide by 30 cm high ridge built to eliminate potential water movement. In intercropping plots, wheat and maize were alternated in 190 cm wide strips, in which, wheat strip was 80 cm wide consisting of six rows with a row space of 12 cm, and maize strip was 110 cm wide consisting of three rows with 40 cm row (Fig. 10). Thus, in the wheat/maize intercropping, wheat account for 42% of the plot area and maize account for 58%. The planting density of sole wheat was 6,750,000 plants ha^−1^ and sole maize was 90,000 plants ha^−1^. For each crop, the same area-based planting density was employed in intercropping and sole cropping. Intercropped wheat was at 2,840,000 plants ha^−1^ and maize was at 52,000 plants ha^−1^. Field maize (*cv. Xian-yu 335*) was planted on 22 April 2018, 22 April 2019 and 20 April 2020, and harvested on 25 September 2018, 22 September 2019 and 25 September 2020. Wheat (*cv. Ning-chun 2*) was sown on 16 March 2018, 17 March 2019 and 17 March 2020 and harvested on July 27, 24 and 27 in 2018, 2019 and 2020. The use of maize and wheat seeds in the present study was permitted by Gansu Agricultural University and it complies with local and national guidelines and legislation. Maize was mulched by plastic film (polyethylene film 0.01 mm thick and 120 cm wide), which made in Lanzhou Green Garden Corporation of China, Lanzhou. It is an innovative technology largely adopted in arid areas to improve maize productivity^52^. There is low precipitation at the testing areas (< 155 mm annually), so that supplemental irrigation was applied. Before soil freezing, 120 mm of irrigation was applied to all plots. Total amount of irrigation was 240 mm for sole wheat, 405 mm for sole maize and 480 mm for intercropping during each growing season. Other agronomic practices, except for the fertilizer application, were kept uniform in this study.

## Plant sampling and analysis

### Aboveground dry matter

The sole and intercropped components were collected for aboveground dry matter determination at 15 days intervals before wheat harvest, and at 20 days intervals after wheat harvest. The first sampling was conducted at 15 days after maize emergence. For the sake of minimizing the influence of destructive sampling on yield formation, 2/3 of the plot in width was used to measure dry matter accumulation, and the remaining 1/3 were used to measure grain yield at physiological maturity. At each sampling date, 20 wheat plants in the same row were randomly selected to determine wheat aboveground dry matter (DM). For maize, 10 individual plants were randomly selected before jointing stage and 5 plants after jointing stage to determine maize DM.

Samples were separated into leaf, stem, and ear of wheat and leaf, stem (include sheath), husk, and ear of maize per plant. All samples were oven-dried at 105 °C for 30 min and weighed after further drying at 80 °C until a constant weight was attained. Finally, the aboveground biomass was used to calculate the transportation amount, and transportation rate of dry matter in vegetative organs to grain, and the contribution rate of vegetative organs to grain according to Yin^50^. The equation was following:

1 $$\begin{array}{*{20}c} {{\text{DTA}} = {\text{LDW}} - {\text{DWM}}} \\ \end{array}$$

2 $$\begin{array}{*{20}c} {{\text{DTR}} = \frac{{{\text{DTA}}}}{{{\text{LDW}}}} \times 100\% } \\ \end{array}$$

3 $$\begin{array}{*{20}c} {{\text{GCR}} = \frac{{{\text{DTA}}}}{{{\text{GDW}}}} \times 100\% } \\ \end{array}$$

where DTA (kg ha^−1^) represents transportation amount of dry matter in vegetative organ, LDW (kg ha^−1^) represents the largest dry weight of the vegetative organ, DWM (kg ha^−1^) represents the dry weight of the same vegetative organ in maturity, DTR represents transfer rate of dry matter in vegetative organ (%), GCR represents contribution rate of vegetative organs to grain (%) and GDW (kg ha^−1^) represents the dry weight of grain.

### Crop growth rate

The crop growth rate was calculated (CGR) (kg ha^−1^ day^−1^) using the following equation:

4 $$\begin{array}{*{20}c} {{\text{CGR}} = \frac{{{\text{W}}_{2} - {\text{W}}_{1} }}{{{\text{T}}_{2} - {\text{T}}_{1} }}} \\ \end{array}$$

where W_2_ and W_1_ are the aboveground biomass accumulation sampled at T_2_ and T_1_.

### Grain yield, biomass yield, yield components, and harvest index

Grain yield (GY) and biomass yield (BY) were measured after air-drying, cleaning of the sole and intercropped systems from all plots. At the maturity stage, 30 wheat plants and 10 maize plants in the undisturbed natural strip were randomly selected to test kernel number per spike (KNS) and thousand-kernel weight (TKW); measure 2.5 × 0.8 m = 2 m^2^ (wheat), 5 × 1.0 m = 5 m^2^ (maize) square area to count the spike number (SN) and calculate the grain yield per unit area by threshing and weighing. Harvest index (HI) was determined by dividing GY by aboveground BY at physiological maturity:

5 $$\begin{array}{*{20}c} {{\text{HI}} = {\text{GY/BY}}} \\ \end{array}$$

### Land use efficiency

The land equivalent ratio (LER) was calculated as follows:

6 $$\begin{array}{*{20}c} {{\text{LER}} = \frac{{{\text{Y}}_{{{\text{im}}}} }}{{{\text{Y}}_{{{\text{sm}}}} }} + \frac{{{\text{Y}}_{{{\text{iw}}}} }}{{{\text{Y}}_{{{\text{sw}}}} }}} \\ \end{array}$$

where Y_im_ and Y_sm_ are the grain yield of intercropped maize and sole maize, respectively, and Y_iw_ and Y_sw_ are the grain yield of intercropped wheat and sole wheat, respectively. A value of LER > 1.0 indicates a yield advantage of intercropping over sole cropping and vice versa.

### Statistical analysis

Data were analyzed at *P* < 0.05 level using Statistical Analysis Software (SPSS software, 21.0, SPSS Institute Ltd, Chicago, USA). Analysis of variance was conducted by using Duncan’s multiple range tests at *P* < 0.05 level to test for the significance of cropping system, N-fertilizer postponed topdressing effects, and their interactions.
